# *Lactiplantibacillus plantarum* KAU007 Extract Modulates Critical Virulence Attributes and Biofilm Formation in Sinusitis Causing *Streptococcus pyogenes*

**DOI:** 10.3390/pharmaceutics14122702

**Published:** 2022-12-02

**Authors:** Irfan A. Rather, Mohammad Younus Wani, Majid Rasool Kamli, Jamal S. M. Sabir, Khalid Rehman Hakeem, Ahmad Firoz, Yong-Ha Park, Yan-Yan Hor

**Affiliations:** 1 Department of Biological Sciences, Faculty of Science, King Abdulaziz University, Jeddah 21589, Saudi Arabia; 2 Center of Excellence in Bionanoscience Research, King Abdulaziz University, Jeddah 21589, Saudi Arabia; 3 Department of Biotechnology, Yeungnam University, Gyeongsan-si 38541, Republic of Korea; 4 Department of Chemistry, College of Science, University of Jeddah, Jeddah 215889, Saudi Arabia; 5 Probionic Corporation, Jeonbuk Institute for Food-Bioindustry, 111-18 Wonjangdong-gil, Deokjin-gu, Jeonju-si 54810, Republic of Korea

**Keywords:** lactobacillus, biofilm, *Streptococcus pyogenes*, virulence attributes

## Abstract

*Streptococcus pyogenes* is one of the most common bacteria causing sinusitis in children and adult patients. Probiotics are known to cause antagonistic effects on *S. pyogenes* growth and biofilm formation. In the present study, we demonstrated the anti-biofilm and anti-virulence properties of *Lactiplantibacillus plantarum* KAU007 against *S. pyogenes* ATCC 8668. The antibacterial potential of *L. plantarum* KAU007 metabolite extract (LME) purified from the cell-free supernatant of *L. plantarum* KAU007 was evaluated in terms of minimum inhibitory concentrations (MIC) and minimum bactericidal concentrations (MBC). LME was further analyzed for its anti-biofilm potential using crystal violet assay and microscopic examination. Furthermore, the effect of LME was tested on the important virulence attributes of *S. pyogenes*, such as secreted protease production, hemolysis, extracellular polymeric substance production, and cell surface hydrophobicity. Additionally, the impact of LME on the expression of genes associated with biofilm formation and virulence attributes was analyzed using qPCR. The results revealed that LME significantly inhibited the growth and survival of *S. pyogenes* at a low concentration (MIC, 9.76 µg/mL; MBC, 39.06 µg/mL). Furthermore, LME inhibited biofilm formation and mitigated the production of extracellular polymeric substance at a concentration of 4.88 μg/mL in *S. pyogenes*. The results obtained from qPCR and biochemical assays advocated that LME suppresses the expression of various critical virulence-associated genes, which correspondingly affect various pathogenicity markers and were responsible for the impairment of virulence and biofilm formation in *S. pyogenes*. The non-hemolytic nature of LME and its anti-biofilm and anti-virulence properties against *S. pyogenes* invoke further investigation to study the role of LME as an antibacterial agent to combat streptococcal infections.

## 1. Introduction

*Streptococcus pyogenes*, a Group A Streptococcus (GAS), is the most frequently isolated pathogen in humans. The infection caused by this pathogenic bacterium can range from mild localized manifestations (tonsillitis and impetigo) to serious systemic diseases, including puerperal sepsis, pneumonia, meningitis, etc. [1,2]. Infections are associated with a high rate of morbidity and mortality on a global scale [3]. Additionally, in predisposed individuals, *S. pyogenes* can induce various acute post-infectious and immune-associated problems, such as severe rheumatic fever, rheumatic heart disease, etc. [4]. *S. pyogenes* produces an arsenal of pathogenic attributes, such as biofilm formation, extracellular proteases, surface-linked M protein, and pyrogenic exotoxins, that allow *S. pyogenes* to establish a broad range of infections in the host [5,6]. The expression of these pathogenicity markers and the acclimatization of *S. pyogenes* to different environmental conditions is coordinated by multifaceted signaling networks, which include different two-component systems (TCS), transcription factors, and gene regulators [7]. More importantly, covRS TCS [8], *Rgg* family of transcriptional factors [9], *srv* [10], and *mga* [11] have been well researched and play a critical role in the regulation of genes associated with *S. pyogenes* virulence. Moreover, antimicrobial resistance among clinical strains of *S. pyogenes* is a major concern and complicates the global therapeutic regimen [12,13,14]. 

Among all reported virulence factors, biofilm formation is the most prominent, as it plays a crucial role in antimicrobial resistance, and pathogenicity prevents the perturbance of therapeutic agents [15]. Biofilms provide extra protection to the bacteria, making them less susceptible to therapeutic drugs and host immune responses. The strategies adopted by the bacterial community include modulation of gene expression, controlled metabolic rate, intercellular communication, composition, and 3D architecture of the extracellular matrix. These are key attributes involved in the virulence of streptococci, thereby resulting in therapy failure and promoting persistent infections [15]. With the rise in multidrug resistance among clinical strains of *S. pyogenes* and the increasing importance of streptococcal biofilms, the discovery of novel anti-virulence agents is of great importance. Contrary to classical drugs, which enforce a selection pressure on microorganisms, the anti-virulent agents directly act on the microbial communication network required for the development of biofilms and establishing infection in the host, thus lowering the probability of resistance development [16]. Although a number of natural products and synthetic compounds have been studied for anti-biofilm properties in *S. pyogenes*, their impact on the virulence attributes have not been explored in detail.

Many food-grade lactic acid bacteria (LAB) have been recognized for their probiotic characteristics and used in some capacity for centuries. Probiotics are live microorganisms giving the host health benefits (direct or indirect) when given in adequate quantity. The health benefits associated with certain fermented foods, generally considered rich LAB sources, have been historically recognized. *L. plantarum* is a Gram-positive LAB species that exhibits ecological and metabolic adaptability and can inhabit a range of ecological niches, including fermented foods, meats, plants, and the mammalian gastrointestinal tract, which are ideal probiotics [17]. In the last decade, researchers have explored various probiotic strains tagged with advanced functional properties and high safety; thus, they have become a desirable goal in medicinal science to naturally restore health, such as through microbiota modulation. In general, antibiotics are the most popular way to prevent and treat bacterial infections [18]; however, the use of these antimicrobials has been a major cause of the onset of antibiotic resistance in many pathogens [19]. LAB are well known for their ability to exert antimicrobial activity against many pathogens [20,21]. Based on many previous studies, LAB have shown antibacterial activity against several pathogens, such as *Clostridium difficile*, *Escherichia coli* [22], *Shigella* spp. [23], *Streptococcus mutants* [24], *Pseudomonas aeruginosa* [25], *Staphylococcus aureus* [26], *S. pyogenes* [27], and also against gastrointestinal and urogenital pathogens [28,29]. However, not much has been explored against oral-pharyngeal pathogens. Furthermore, the mechanisms behind the anti-virulent and anti-biofilm properties of LAB and their impact on the expression of genes associated with various pathogenic attributes are not well understood. Thus, this study aims to discover a suitable LAB strain with anti-virulent and anti-biofilm properties against the sinusitis-inducing pathogen *S. pyogenes*.

## 2. Materials and Methods

### 2.1. Identification and Characterization of the Lactobacillus Strains

*Lactiplantibacillus plantarum* KAU007 was isolated from camel milk in Jeddah, Saudi Arabia. The isolation and molecular characterization followed the same protocol as described elsewhere [30]. In brief, fresh milk samples were directly collected from the udder of the camel. The milk samples were collected in sterile tubes and transported to the laboratory within two hours, after being stored in an icebox. In addition, milk samples were diluted (1–10) to isolate beneficial bacteria using PCB media and de Man Rogosa agar plates. In the end, selected isolates were sent to Macrogen for 16S rRNA gene sequencing (Seoul, Republic of Korea).

### 2.2. Growth Conditions of S. pyrogens

In the present study, *S. pyogenes* ATCC 8668 was used for all the assays. The bacteria were regularly maintained on tryptose agar (Sigma-Aldrich, St. Louis, MO, USA). For biofilm formation and other quantification assays, Todd Hewitt broth (Sigma-Aldrich, USA) was supplemented with 0.5% yeast extract and 1% glucose (THYG). To prepare the standard inoculum suspension, overnight-grown *S. pyogenes* at 10^5^ CFU/mL concentration was used.

### 2.3. Revival of L. plantarum KAU007 

*L. plantarum* KAU007 was revived from the glycerol stock on MRS agar plates (30 °C for 48–72 h). A single colony was inoculated in MRS broth and incubated at 30 °C for 24 h, followed by centrifugation (8000× *g* for 10 min) and washing of the cell pellet with PBS. The pellet was dissolved in PBS, and the cell concentration was adjusted to 0.5 McFarland standard (1.5 × 10^8^ CFU/mL) by using a turbidimeter. Later, this was used as a standard inoculum for secondary metabolite preparation.

### 2.4. Preparation of L. plantarum KAU007 Metabolite Extract

The method described by Badwaik et al. [31] was adopted to produce secondary metabolites. Briefly, the MRS broth was inoculated with a standard inoculum of *L. plantarum* KAU007 (2% *v*/*v*) and kept at 30 °C for 48 h. Afterward, the fermented media was centrifuged (4400 rpm for 20 min at 4 °C), and the top supernatant was isolated as the cell-free supernatant (CFS). The CFS was then filtered using a 0.22 μm vacuum filtration system (Nalgene, Rochester, NY, USA) to remove any remaining cells. The secondary metabolites from the CFS were then extracted in an equal ratio using ethyl acetate (EA; Sigma-Aldrich, USA). Post-extraction, organic, and interfacial layers were collected and evaporated using a rotary vacuum evaporator (Buchi, Essen, Germany). A working solution of *L. plantarum* KAU007 metabolite extract (LME; 10 mg/mL) was prepared in 1% dimethyl sulfoxide (DMSO) and was used to evaluate antimicrobial activity and anti-biofilm activity.

### 2.5. Estimation of Antibacterial Activity of LME 

The antibacterial activity of LME was evaluated by determining the minimum inhibitory concentrations (MIC) against *S. pyogenes* ATCC 8668. The broth microdilution assay using Brain-Heart Infusion broth by following the method previously described in the Clinical Laboratory Standards Institute (CLSI) guidelines [32]. Briefly, the tested concentration of all the LMEs ranged from 2500 to 1.22 µg/mL. The lowest concentration of LMEs that inhibited visible *S. pyogenes* growth was considered their MIC. Furthermore, erythromycin (0.1–0.031 µg/mL) was used as a positive control in the experiment. Following MIC, the minimum bactericidal concentration (MBC) of all the LMEs against *S. pyogenes* was determined. The bactericidal activity was evaluated by spotting 30 μL from the wells beyond MIC value on BHI agar plates and incubating for 24 h at 37 °C. The lowest concentrations of LMEs that destroyed around 99.9% of *S. pyogenes* cells were considered MBC [33]. 

### 2.6. Estimation of Anti-Biofilm Property of LME against S. pyogenes

Various concentrations of LME (78.12 µg/mL–1.22 µg/mL) were used to evaluate its effect on *S. pyogenes* biofilms, and the method previously described was adopted for this experiment [34]. Briefly, LME was added to respective wells of a 24-well microtiter plate containing 1 mL of THYG broth to achieve the desired concentrations. Later, each well was supplemented with 1% of standard inoculum suspension (0.5 McFarland standard), followed by incubation at 37 °C for 24 h without shaking. Post-incubation, planktonic cells were aspirated, wells were gently washed with PBS, and adhered cells were stained with 0.4% crystal violet for 10 min. Thereafter, the cells were washed with PBS to remove excess stain from the wells, and the cells were allowed to dry. The bound crystal violet was extracted using 20% glacial acetic acid, and absorbance was recorded at 570 nm. The total amount of cells attached in the form of biofilm to the wells of the microtiter plate was directly proportional to the amount of dye extracted from the wells. The percent biofilm inhibition was calculated using the following formula. The lowest concentration showing ≥90% biofilm inhibition was considered to be the biofilm inhibitory concentration (BIC).

(1) $$\%\;\mathrm{biofilm}\;\mathrm{inhibition}=\left[\frac{\mathrm{control}\;\mathrm{OD}\;\mathrm{value}-\mathrm{treated}\;\mathrm{OD}\;\mathrm{value}}{\mathrm{control}\;\mathrm{OD}\;\mathrm{value}}\right]\times 100$$

### 2.7. Effect of LME on S. pyogenes Growth 

To understand the effect of LME on the growth of *S. pyogenes*, the cell density measurements were quantified using the absorbance method measured at a different timeline. Briefly, LME at its BIC and sub-BIC values were individually added to 100 mL of THYG broth supplemented with 1% standard inoculum suspension. The mixture was inoculated at standard conditions (37 °C and 150 rpm), and the growth dynamics of treated and untreated samples were compared by recording absorbance at 600 nm at predetermined time points (every 2 h up to 24 h) [35].

### 2.8. Light Microscope Micrograph

The anti-biofilm effect of LME was further confirmed with the help of light microscopy; the *S. pyogenes* biofilms were allowed to grow on glass coverslips placed in 24-well microtiter plate, both in the presence and absence of LME (at BIC) under biofilm-forming conditions (37 °C for 24 h without shaking). Then, the coverslips were gently washed with PBS and stained with 0.4% crystal violet for 10 min; excess stain was removed by washing, air dried, and observed under a light microscope (Leica DM 500 microscope), and pictures were captured with an attached digital camera.

### 2.9. Effect of LME on Secreted Protease Activity in S. pyogenes

The effect of LME on secreted protease activity in *S. pyogenes* was evaluated on tryptose agar (Sigma-Aldrich, USA) containing 1% of skim milk powder (Sigma-Aldrich, USA). The standard inoculum suspension was exposed to LME (at BIC value) for 4 h; after that, the culture was centrifuged and washed with PBS and spotted (20 μL) onto the agar plate, whereas untreated *S. pyogenes* was used as a negative control for the experiment. The plates were incubated at 37 °C for 24 h, and a zone of clearance was recorded around the *S. pyogenes* growth [9].

As previously suggested, the quantification of secreted protease was performed in liquid culture [36]. Briefly, the *S. pyogenes* were grown with and without LME (at BIC value) under standard conditions (37 °C and 24 h). The cell-free supernatant was obtained by centrifuging (5000 rpm 15 min) culture broth, collecting the cell-free media in a sterile tube, and aliquots (1 mL) of secured supernatant to quantify the presence of secreted protease in the cell-free broth. The aliquots were mixed with activation buffer [200 μL; 1 mM EDTA and 20 mM DTT in 0.1 M sodium acetate buffer (pH 5.0)] followed by incubation at 40 °C for 30 min. After that, 2% azocasein (400 μL) was added, the mixture was again incubated for 1 h at room temperature, and then the reaction was terminated by mixing trichloroacetic acid (10%; 20 min incubation at room temperature). Later, the tubes were centrifuged at 3000 rpm for 5 min, and the supernatant was collected in a sterile tube and mixed with an equal volume of NaOH (1 M). The absorbance was recorded at 440 nm.

### 2.10. Effect of LME on the Hemolytic Property of S. pyogenes

The anti-hemolytic property of LME was determined on agar supplemented with horse blood as described elsewhere, with some modifications [16] with some modifications. Briefly, the horse blood was diluted in autoclaved THYG broth to a final concentration of 2% (*v*/*v*). Different experimental sets were prepared for analysis, (a) standard inoculum suspension (10%) of *S. pyogenes* and DMSO was added to the diluted horse blood; (b) standard inoculum suspension (10%) of *S. pyogenes* and LME (at BIC value) was added to the diluted horse blood; (c) LME (at BIC value) alone was added to the diluted horse blood and was referred as blank. All the different sets were incubated at 37 °C for 1 h, followed by incubation at 4 °C for 1 h. Later, tubes were centrifuged at 5000 rpm for 10 min; supernatants were secured, and absorbance was recorded at 405 nm.

### 2.11. Effect of LME on the Production of an Extracellular Polymeric Substance in S. pyogenes

The impact of LME on the synthesis of extracellular polymeric substances (EPS) in *S. pyogenes* was quantified by estimating the total carbohydrate covering the bacterial cells based on the method as previously described [7]. Briefly, the *S. pyogenes* was propagated with and without LME under standard growth conditions. Afterward, the culture broth was centrifuged (10,000 rpm for 10 min), and the pellet was washed and resuspended in PBS (200 μL). Phenol (5%) was added in equal volumes to the resuspended pellet, followed by the addition of concentrated H_2_SO_4_ containing hydrazine sulfate (0.2%), mixed well, and incubated for 1 h at room temperature away from light. The samples were spun for 10 min (10,000 rpm), and absorbance was recorded at 490 nm.

### 2.12. Effect of LME on Microbial Adhesion to Hydrocarbon

The impact of LME on the hydrophobicity of the cell surface in *S. pyogenes* was evaluated using the microbial adhesion to hydrocarbon (MATH) assay as previously described [37]. Briefly, the bacterial cells were grown with and without LME (at BIC value), and the culture was washed and diluted in PBS to obtain 0.3 OD at 600 nm. The culture was mixed with toluene in equal ratio, vortexed (2 min), and kept undisturbed at room temperature to allow phase separation. Afterward, the aqueous phase was separated, and absorbance was recorded at 600 nm. *S. pyogenes* grown in the presence of DMSO alone was used as a negative control. The percentage of cell surface hydrophobicity was calculated by using the below formula.

(2) $$\%\;\mathrm{cell}\;\mathrm{surface}\;\mathrm{hydrophobicity}=1-\left[\frac{\mathrm{OD}600\;\mathrm{nm}\;after\;vortexing}{\mathrm{OD}600\;\mathrm{nm}\;\mathrm{before}\;\mathrm{vortexing}}\right]\times 100$$

### 2.13. Real-Time PCR Analysis 

Real-time qPCR was performed to analyze the effect of LME on the expression profile of genes associated with biofilm formation and virulence. The total RNA of *S. pyogenes* grown with and without LME (at BIC) for 24 h was extracted using an RNA MiniPrep kit (Inqaba Biotechnical Industries Ltd., Pretoria, South Africa) based on the protocol as suggested by the manufacturer. The concentration of the RNA extracted was measured using Nanodrop 2000 spectrophotometer (Thermo Scientific, Waltham, MA, USA). Following RNA extraction, cDNA was synthesized by kit (Lasec South Africa Ltd., Cape Town, South Africa) according to the manufacturer’s protocol. The qPCR was performed using PowerUp^TM^ SYBR^TM^ Green Master Mix (Applied Biosystems) in a RocheLight^®^ Cycler Nano instrument Real-time PCR system (Roche, Basel, Switzerland) by following the thermal cycling conditions previously described [7]. Briefly, the following thermal cycling conditions for all RT-qPCR reactions were used; UDG activation at 50 °C for 2 min (Hold), Dual-lock DNA polymerase at 95 °C for 2 min (Hold), 40 cycles of denaturation at 95 °C for 15 s, annealing at 46 °C for 15 s, and extension 72 °C for 1 min. Dissociation curve conditions (melt curve stage) were as follows: Pre-melting at ramp rate of 1.6 °C/s, 95 °C and 15 s; Melting at ramp rate of 1.6 °C/s, 60 °C and 1 min; Melting at ramp rate of 0.15 °C/s, 95 °C and 15 s. The gene expression profile of candidate genes was normalized to the housekeeping genes *gyrA*. The oligonucleotide sequences used in the study are shown in Table 1.

### 2.14. GC-MS Analysis 

The GC-MS study was carried out using an Agilent 7890/M780EI gas chromatograph (GC, Agilent Technologies, Palo Alto, CA, USA) linked with a PERSEE mass spectrometer (MS, Shimadzu, Kyoto, Japan) and an Agilent AS-2912 autosampler. In a split ratio of 8:1, 1.0 mL of sample was injected into a deactivated and fused-silica Agilent DB-5MS capillary column (30 m × 0.25 mm × 0.25 μm film, Agilent J&W Scientific, Folsom, CA, USA). After the column preparation, the parameters such as the electron impact (EI) ion source temperature and electron beam were set to 250 °C and 70-eV, respectively. Following this, the injector temperature was maintained at 280 °C with a detector voltage of 0.96 kV and a solvent delay of 6 min. The flow rate of helium carrier gas (99.999%) was set to 1.0 mL/min. A GC oven was maintained at 90 °C for 3 min and ramped at 3 °C/min to 160 °C, then ramped at 2 °C/min to 220 °C, where it was held for 1 min, then ramped at 10 °C/min to 290 °C. The masses were acquired in full scan mode with a scanning velocity of 0.2 s between 45 and 550 *m*/*z*.

### 2.15. Statistical Analysis

All the experiments were carried out in triplicates at least two times, and the results were presented as average ± standard error of the mean. Statistical analysis was conducted in Graph Pad Prism version 9.1.0 by utilizing Student’s unpaired two-tailed *t*-test. The *p* value < 0.05 was considered statistically significant.

## 3. Results and Discussion 

### 3.1. Antibacterial Activity of LME against S. pyogenes

The metabolite extract of L. plantarum KAU007 was extracted in organic solvents, as this technique gives a higher yield compared to other solvents [50]. Ethyl acetate was used for the extraction of the metabolite from the cell-free supernatant of *L. plantarum*, and the final yield was 0.11 g/mL. The extract was found to be active against planktonic cells of *S. pyogenes*. The LME was found to inhibit growth and survival at very low dosage (MIC, 9.76 μg/mL; MBC, 39.06 μg/mL), whereas the MIC and MBC values of erythromycin against *S. pyogenes* were recorded as 0.12 and 0.25 μg/mL, respectively. 

The present investigation focused on the effectiveness of LME’s antibacterial activity against GAS. LAB are a group of heterogeneous bacteria, and their antibacterial activity and virulence mechanisms against different pathogens have been widely explored [51,52,53]. Additionally, in vitro research has shown antibacterial activity of lactobacilli against *C. difficile*, *E. coli*, *Shigella species*, *S. mutans*, *P. aeruginosa*, and *S. aureus* [22,23,24,25,26,54]. Similarly, the inhibitory effect of lactobacilli against *S. pyogenes* has been well reported in the literature [27,52], and the present finding is in agreement with previous work. Although in vitro activity cannot fully reflect in vivo efficacy, our results demonstrate the inhibitory activity of LMEs and their potential in treating *S. pyogenes*-associated infections. However, further animal experiments are required to prove our hypothesis. LME was further analyzed to understand its impact on various virulence attributes in *S. pyogenes* and the probable mode of antibacterial action.

### 3.2. Inhibition of Biofilm Formation by LME without Hampering the Growth of S. pyogenes

**Figure 1:**
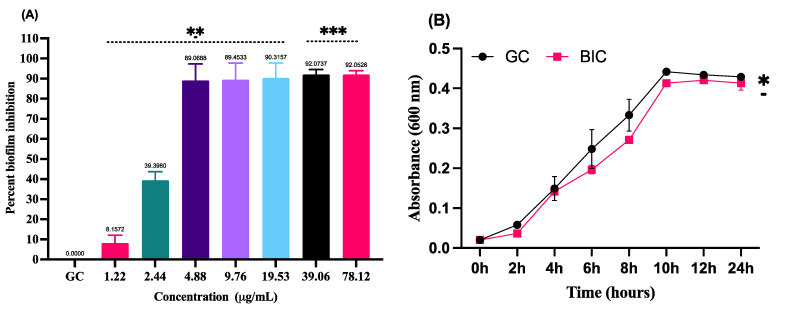
(**A**) Percent biofilm inhibition in *S. pyogenes*. The inhibition of biofilm formed by *S. pyogenes* in presence of LME at various concentrations. GC, growth control. *** *p* value = 0.0002, ** *p* value = 0.0042. (**B**) Growth curve dynamics. Figure represents the effect of LME (at BIC value) on the growth curve of *S. pyogenes*. * *p* value = 0.014.

The anti-biofilm potential of LME was evaluated by microtiter plates using crystal violet assay, whereas the effect of LME (at BIC value) on the growth dynamics of *S. pyogenes* was confirmed by spectrophotometrically monitoring the bacterial growth (2 h intervals). The obtained data represented a concentration-dependent anti-biofilm activity of LME where, at a low concentration of 4.88 μg/mL (0.5 × MIC), the percent biofilm inhibition was 89.92%; additionally, at higher concentrations (78.12 and 39.06 μg/mL) the maximum inhibition obtained was 92%. Therefore, 4.88 μg/mL was considered as the BIC value which was used for further analysis (Figure 1A). The anti-bactericidal effect of LME at the BIC value was confirmed by the growth curve, where no deviation in the bacterial growth dynamics was observed in the absence and presence of LME (Figure 1B). 

**Figure 2:**
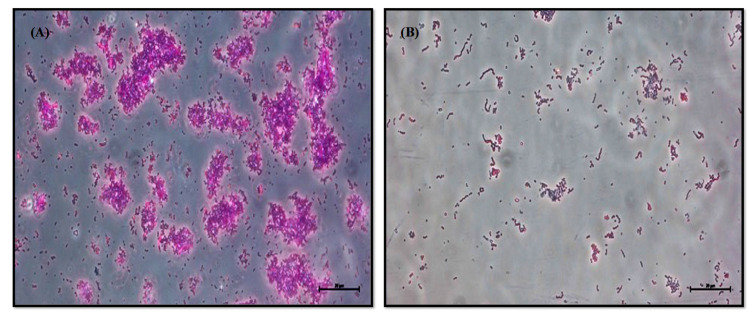
Light micrograph of biofilm formed by *S. pyogenes*. The figure displays, (**A**) the control *S. pyogenes* forming a well—structured biofilm stained with crystal violet. (**B**) LME (4.88 μg/mL) impedes microcolony and biofilm formation in *S. pyogenes*.

The in-depth analysis of the anti-biofilm property of LME was conducted using a light microscope. The untreated bacterial cells displayed dense and well-organized microcolonies scattered throughout the coverslip; on the other hand, the biofilm architecture in the treated samples was scarce, and very few cells were found to adhere to the surface (Figure 2). Furthermore, the images showed the abundance of extracellular polymeric substances (EPS) in the cellular aggregates of untreated control biofilms, whereas scattered bacterial chains with clear outer surface showing decreased EPS production were observed in the sample treated with LME (4.88 μg/mL; BIC value).

The non-bactericidal anti-biofilm property of LME against *S. pyogenes* has the potential to be a perfect anti-biofilm agent because it will minimize the chance of resistance development in *S. pyogenes* compared to commonly used antibiotics. Additionally, the light microscopy images of the biofilms advocated the significant decrease in biofilm formation with very few bacterial chains adhered to the cover slip. This feature of LME may have a crucial impact on clinical settings, because it is challenging to treat biofilm- associated infections caused by *S. pyogenes*. The inhibitory properties of different LAB-based probiotics have been established against *S. pyogenes* [55], a broad range of oral pathogenic biofilms [56], and the most common multidrug-resistant urogenital pathogens [57]. Thus, our findings are in alignment with the previous results and further ensure the anti-biofilm characteristics of LME against *S. pyogenes*.

### 3.3. LME Mitigates Production of Secreted Protease in S. pyogenes 

**Figure 3:**
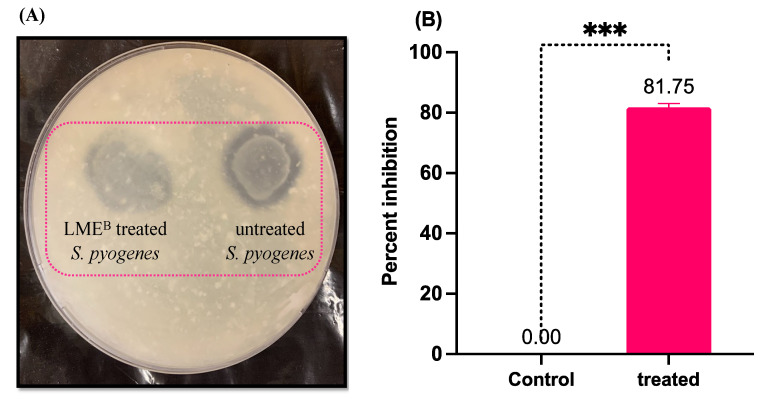
Effect of LME on extracellular protease production in *S. pyogenes*. (**A**) The picture shows the diminished zone of proteolysis around *S. pyogenes* growth in tryptose agar medium (1% skim milk) when treated with LME. (**B**) The percent inhibition of extracellular protease production in treated and untreated *S. pyogenes*. *** *p* value = 0.0001.

Cysteine proteases are the most important secreted protease in *S. pyogenes*; therefore, the tendency of LME to inhibit its production in *S. pyogenes* was both qualitatively and quantitatively evaluated. Compared to the untreated control, the zone of clearance produced by the treated bacteria was significantly low (Figure 3A). The quantitative analysis was conducted to further confirm the impact of LME in inhibiting the production of secreted protease in *S. pyogenes*. For this purpose, azocasein was used as a substrate, and the amount of dye released in the suspension was directly proportional to the amount of protease production. The data obtained reflected an 81.75% reduction in protease production in LME-treated *S. pyogenes* (Figure 3B).

The reduced protease production quantified by azocasein assay and lower enzymatic activity on a tryptose agar plate in LME-treated *S. pyogenes* further ascertained the capability of LME to downregulate speB in *S. pyogenes*. The reduced activity and production of secreted streptococcal cysteine protease in treated *S. pyogenes* drastically reduced the ability of bacteria to damage the host matrix proteins, manipulating host immunity and, therefore, compromising its potency to cause tissue damage and infection dissemination.

### 3.4. LME Mitigates Hemolysin Production in S. pyogenes 

**Figure 4:**
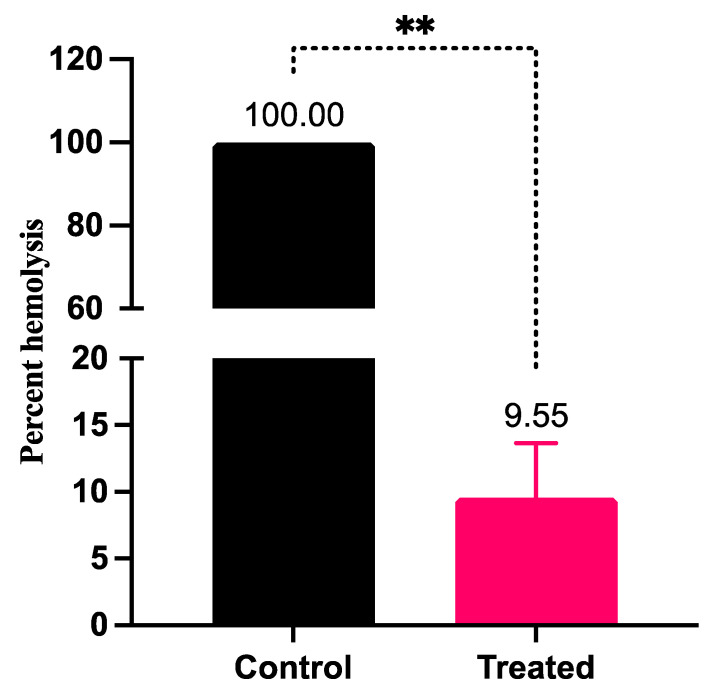
Effect of KAU007 on hemolytic property of *S. pyogenes*. The figure represents the impact of LME on percent streptococcal hemolysis ** *p* value = 0.001.

The effect of LME on the production of hemolysin was quantitatively estimated by measuring the amount of hemoglobin liberated after the breakdown of red blood cells by *S. pyogenes* in the presence and absence of LME. Treatment with LME drastically reduced the hemolytic property of *S. pyogenes* compared to the untreated control. The percent hemolysis was lowered to 9.55%, whereas the percent hemolysis was 100% in untreated *S. pyogenes* (Figure 4).

### 3.5. LME Abrogates the EPS Production and Cell Surface Hydropgobicity in S. pyogenes 

**Figure 5:**
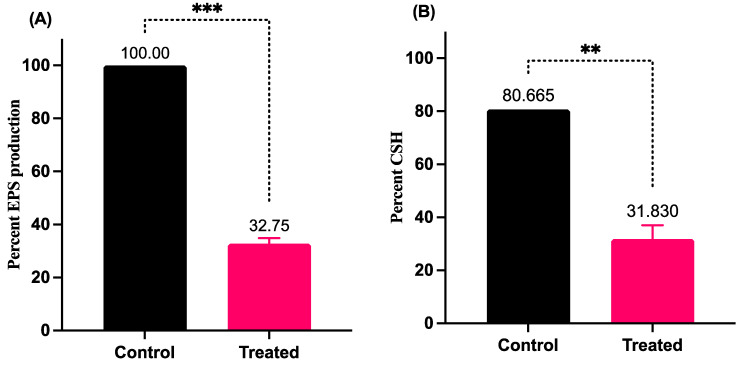
Effect of LME on EPS production and cell surface hydrophobicity (CSH). The figure demonstrates the modulation of EPS production (*** *p* value = 0.0005) and CSH (** *p* value = 0.0056) in the presence (treated) and absence (control) of LME.

LME inhibted EPS production in *S. pyogenes*; EPS was lowered to 32.5% compared to the untreated control (Figure 5A). Similarly, LME resulted in a reduction of cell surface hydrophobicity in *S. pyogenes*. The untreated control displayed 80.66% cell surface hydrophobicity, whereas the LME-treated bacteria showed only 31.83% cell surface hydrophobicity (Figure 5B). 

The impact of LME on cell surface hydrophobicity advocates its anti-biofilm properties against *S. pyogenes* because the ability of *S. pyogenes* to form biofilm starts with the initial attachment phase. 

### 3.6. LME Modulates Expression of Biofilm and Virulence-Associated Genes in S. pyogenes 

**Figure 6:**
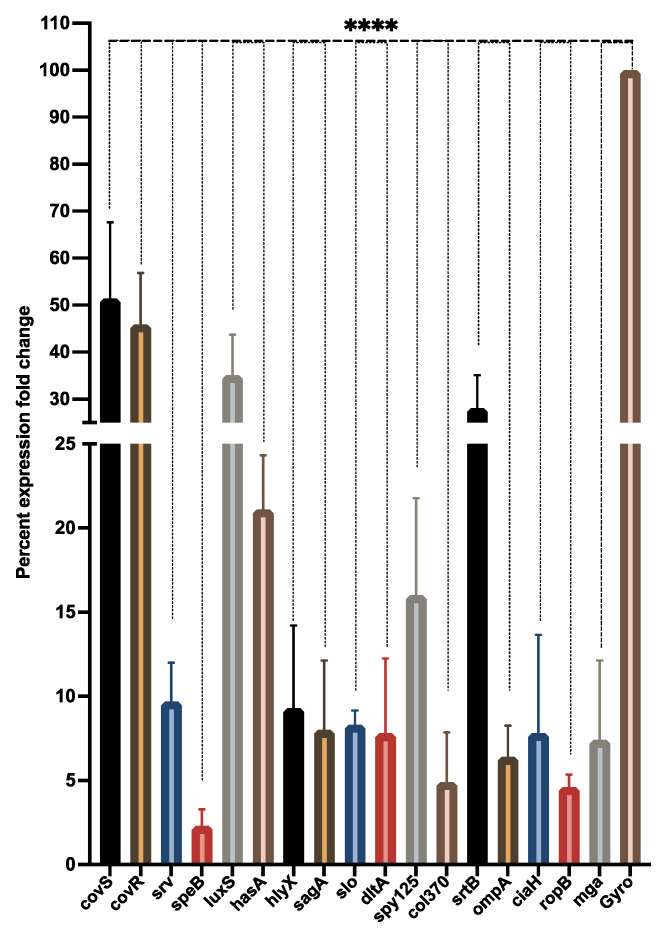
Percent expression fold change in biofilm and virulence-associated genes of *S. pyogenes*. The qPCR analysis demonstrated the modulatory effect of LME on the expression profile of target genes associated with biofilm and virulence in *S. pyogenes*. **** *p* value < 0.0001.

The LME significantly impacted (4.88 μg/mL) the expression of biofilm and virulence-associated genes in *S. pyogenes* (Figure 6). Compared with the housekeeping gene (gyrA), for which the percent expression fold change was 100%, all the target genes were downregulated in the treated sample. A remarkable downregulation was observed in some genes, namely srv, speB, hlyX, sagA, slo, dltA, col370, ompA, ciaH, ropB, and mga, where the percent expression fold change was below 10%. Whereas, covR, covS, luxS, hasA, spy125, and srtB were moderately downregulated with a percent expression fold change of 51.39%, 45.86%, 35.06%, 21.08%, 15.98%, and 28.14%, respectively.

The qPCR analysis of the genes associated with pathogenicity and biofilm formation was conducted to uncover the probable mode of action of LME against biofilm inhibition and other virulence factors.

All the target genes studied were downregulated in the treated sample, suggesting the impact of LME on pathogenicity of *S. pyogenes* at the molecular level. The ropB is considered to be a universal transcriptional regulator associated with bacterial metabolism, stress response, and virulence [58]. Furthermore, speB (streptococcal erythrogenic toxin B; secreted streptococcal cysteine protease) is responsible for tissue damage and the dissemination of infection [10]. The srv (streptococcal regulator of virulence) has been found to be associated with the expression of speB, and the downregulation of srv resulted in the downregulation of speB in the treated sample. The dltA is involved in the process of D-alanylation of lipotheichoic acid (LTA), which is mainly responsible for the cell surface hydrophobicity in *S. pyogenes* [59] and its mutation can lead to lack of D-alanine in LTA, resulting in reduced biofilm formation in various bacterial species [60]. Thus, clarifying the anti-biofilm property of LME against *S. pyogenes*. The cell surface hydrophobicity quantification further demonstrated that LME treatment strongly modulates the hydrophobicity of *S. pyogenes*. The genes col370 (promote adhesion to epithelial cells), mga, and ompA, play an important role in the biofilm formation in many bacterial isolates [45,47,48], and thus, support the present finding in which their downregulation reduced the biofilm formation in treated *S. pyogenes*. 

In addition, the real-time PCR results also demonstrated the significant downregulation of genes which are critical for hemolysis, namely, sagA (responsible for streptolysin S synthesis), slo (responsible for streptolysin O synthesis), and hlyX (hypothetical protein responsible for hemolysis) upon LME treatment. This result was in agreement with the decrease in percent hemolysis as observed in hemolysin assay upon LME treatment. 

The genes covR and covS are involved in the covRS TCS pathway, and have been well studied for their role in regulating genes, including luxS (involved in quorum-sensing system), mga (a stand-alone regulator), and hasA (participant in hyaluronic acid production) [8]. Furthermore, the antioxidant property of LME has been uncovered by the real-time PCR results, where ciaH, responsible for oxidative stress response [49], was downregulated, suggesting the impact of LME oxidative stress in *S. pyogenes*. In the present study, all these genes were downregulated, suggesting the impact of LME on several crucial metabolic processes in *S. pyogenes*. 

Additionally, the role of srtB has been associated with the growth pattern in *S. pyogenes* and its mutation, resulting in an aggregated pattern of growth in liquid medium [46]. The treatment with LME resulted in the downregulation of srtB, which further indicates the formation of big bacterial clumps, facilitating its detection by the host innate immune system and triggering its elimination when compared to individual cells [34,61]. This gives a clear indication that LME-induced bacterial aggregation may increase the chance of pathogen recognition and clearance by the host immune system.

### 3.7. Chemical Composition of LME

**Figure 7:**
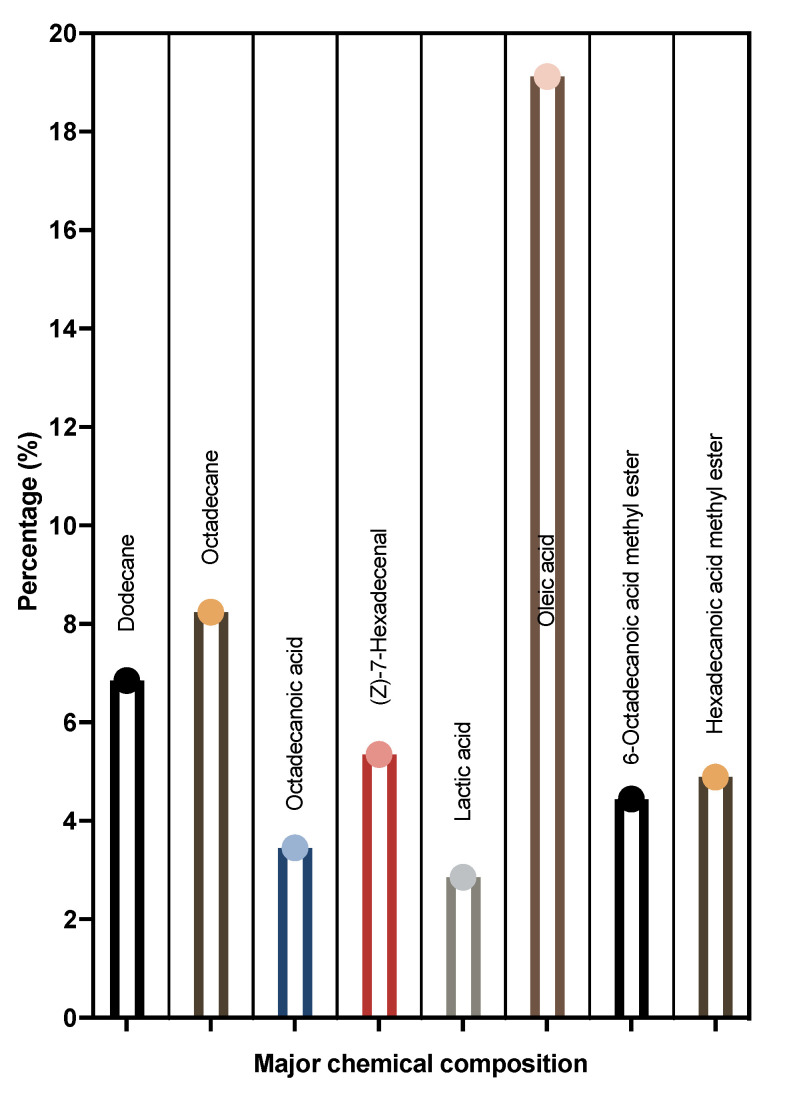
Major composition of the metabolites obtained from LME.

The GC-MS analysis of LME results in the identification of various compounds. The major composition of LME is shown in Figure 7. The metabolic profile obtained from the typical GC-MS total ion chromatogram (TIC) is illustrated in Figure S1. From the TIC, 32 endogenous metabolites were identified, with major components being long-chain saturated fatty acids, hydroxy acids, long-chain carboxylic acid derivatives, aliphatic hydrocarbons, and some miscellaneous compounds in small proportions, as shown in Table S1.

## 4. Conclusions

The current results demonstrated the potential anti-virulent property of LME, highlighting its effectiveness on the biofilm formation and various virulence attributes in *S. pyogenes*. The obtained data suggested that LME targets multiple genes responsible for biofilm formation and pathogenicity markers in *S. pyogenes*. This modulated extracellular protease production, cell surface hydrophobicity, EPS production, and hemolysis. The decrease in cell surface hydrophobicity affects the initial adhesion step, which is crucial for the biofilm formation cascade, and was believed to be the main reason for the biofilm inhibition. At the same time, a remarkable decrease in the synthesis of speB and hemolysin was considered to be the cause of the compromised virulence. Therefore, the current study demonstrates the anti-virulence potential of LME against *S. pyogenes*, and determines there to be much promise in terms of therapeutics. However, in vivo studies are required to further enlighten the anti-biofilm and anti-virulence properties of LME against *S. pyogenes*.

## Figures and Tables

**Table 1:** Candidate genes, their role and primer sequences.

Gene	Purpose	Primer Sequence		Source
		Forward	Reverse	
*covS*	Senor kinase gene of covRS TCS. Regulates biofilm and virulence	GAGTGAGCGCGATATCACAA	GCAAGCCAGGAGATGATTCT	[8]
*covR*	Repressor gene of covRS TCS	TGCGCGTGATTCTATTATGG	GGCGGAAAATAGCACGAATA	[8]
*srv*	Biofilm formation and virulence factor regulation	CGGCATTGTGAAACAGAGTG	TCTGACTCGATGCGAACATT	[38]
*ropB*	Global transcriptional factor. Regulates stress, metabolism and virulence	TGATATGGATACGGCAAAACA	TTGACCAAGGCAAAAAGGTT	[39]
*speB*	Extracellular cysteine protease production	CTAGGATACTCTACCAGCG	CAGTAGCAACACATCCTG	[40]
*luxS*	Involved in quorum sensing	CTTTTGGCTGTCGAACAGGT	TCCAGGAACATCTTCCCAAG	[41]
*mga*	Virulence factor regulation	GATCCGTTACTACAAGGG	GTTACTTGTCTGCCTCCT	[11]
*hasA*	Hyaluronic acid capsule synthesis	AGCGTGCTGCTCAATCATTA	AACATCGATCATCCCCAATG	[41]
*hlyX*	Hemolyisn production	GCGCAATACCCAAAATCAGA	CGATTTCACCGACGATTTCT	[42]
*sagA*	Streptolysin S production	AAACAACTCAAGTTGCTCCTG	TGGCGTATAACTTCCGCTAC	[43]
*slo*	Streptolysin O production	GCCAATGTTTCAACAGCTATTG	CGGAGCTGCACTAAAGGCCGC	[42]
*dltA*	D-alanylation of Lipotheicoic acid	GCATTTGGACATCGACTCCT	GTTTTCGAGCCGTAGAAACG	[44]
*spy125*	Minor pilin subunits on cell surface	AGAGATTAGCGACGCAACAG	ATGGCCATATGTCTCCACCA	[45]
*col370*	Collagen-like surface protein involved in adhesion	AACCCAGATACTGCACCACA	GCGAGCTGATTACCACCTTG	[45]
*srtB*	Class C sortase production involved in aggregation	GCTGGTTTTGGTTTGTGGGA	CCCCGGGATATTTAACCAACC	[46]
*ompA*	Outer membrane protein involved in biofilm and stress response	GTGCTTCCTGGCTATGAACC	GCAGCGGGTTGGTTATTGTA	[47,48]
*ciaH*	Antioxidant stress response TCS	GGCGGTCTTACAGAATCGTC	CATGTTGCGAACCTCGTCTA	[49]
*gyrA*	Gyrase production (housekeeping gene in the present study)	CAACGCACGTAAGGAAGAAA	CGCTTGTCAAAACGACGTTA	

## Data Availability

The data created are cited in the manuscript and provided as supplementary.

## Supplementary Materials

The following supporting information can be downloaded at: https://www.mdpi.com/article/10.3390/pharmaceutics14122702/s1, Figure S1: Total ion chromatogram (TIC) of LME; Table S1: Chemical composition of the metabolites obtained from LME.
