# Supplementary material for: Lactiplantibacillus plantarum KAU007 Extract Modulates Critical Virulence Attributes and Biofilm Formation in Sinusitis Causing Streptococcus pyogenes
Source: Pharmaceutics. 2022 Dec 2;14(12):2702. doi: 10.3390/pharmaceutics14122702 (PMC9780990; doi:10.3390/pharmaceutics14122702)
Supplement: Supplementary file 1 [file pharmaceutics-14-02702-s001.zip › pharmaceutics-2010904-supplementary.pdf]

Supplementary

Lactiplantibacillus plantarum KAU007 *extract modulates critical virulence attributes and biofilm formation in sinusitis causing Streptococcus pyogenes*

Irfan A. Rather[1,2,3,*], Mohammad Younus Wani[4], Majid Rasool Kamli[1,2], Jamal S.M. Sabir[1,2], Khalid Rehman Hakeem[1], Ahmad Firoz[1], Yong Ha Park[3,5], Yan Yan Hor[3,*]

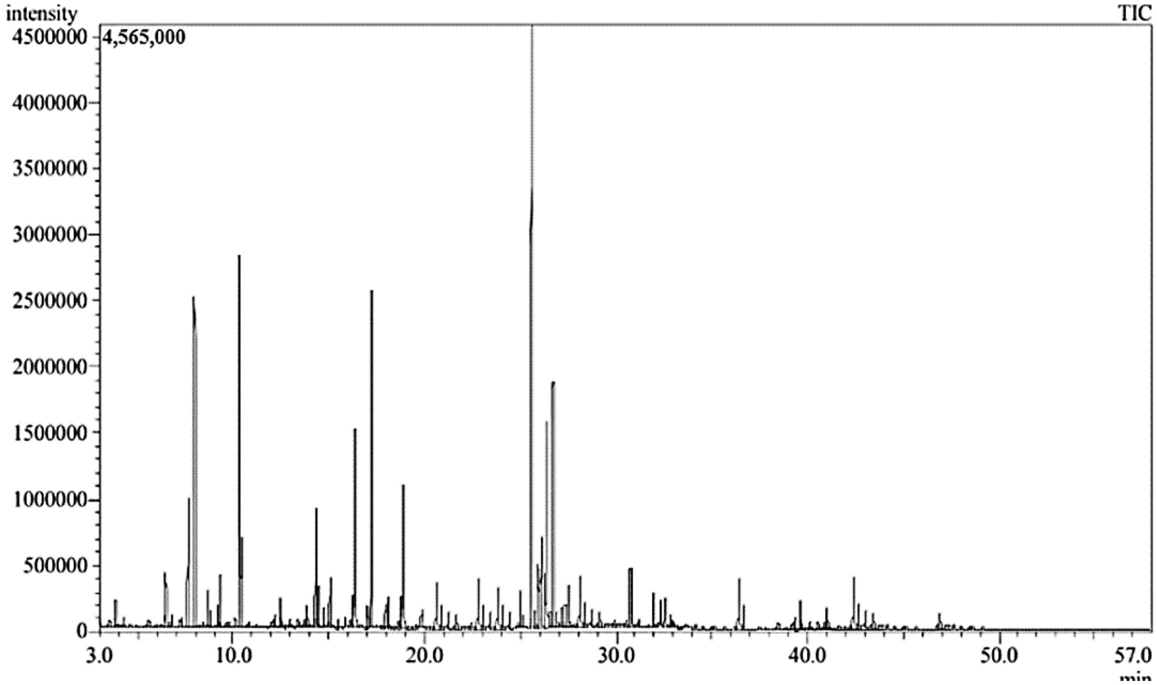

Figure S1. Total ion chromatogram (TIC) of LME.

Table S1: Chemical composition of the metabolites obtained from LME.

S.No.	Metabolite[#]	M.Wt	Retention time	Percentage
**1**	Tetradecane	226.41	6.713	1.85±0.016
**2**	Pentadecane	198.39	7.857	2.95±0.048
**3**	Hexadecane	170.33	7.901	0.94±0.016
**4**	**Dodecane**	**252.5**	**7.958**	**6.85±0.028**
**5**	9-octadecane	266.5	9.293	1.75±0.089
**6**	**Octadecane**	**252.5**	**10.727**	**8.24±0.048**
**7**	Nonadecane	212.4	14.533	2.75±0.040
**8**	2-Methyldecane	156.3	15.466	1.87±.032
**9**	**Octadecanoic acid**	**284.48**	**16.331**	**3.45±0.028**
**10**	**(Z)-7-Hexadecenal**	**238.41**	**17.231**	**5.35±0.028**
**11**	3-hydroxybutyric acid	104.10	18.133	1.60±0.016
**12**	**Lactic acid**	**90.08**	**18.966**	**2.85±.032**
**13**	Sebacic acid	202.25	19.931	0.85±0.040
**14**	Benzoic acid	60.05	20.902	1.88±0.040
**15**	Acetic acid	132.15	22.876	1.96±0.097
**16**	Propanoic acid	118.13	24.075	1.45±0.048
**17**	4-aminobutyric acid	103.12	25.069	1.43±0.048
**18**	**Oleic acid**	**282.47**	**25.513**	**19.12±0.032**
**19**	Stearic acid	284.48	25.851	2.86±0.016
**20**	**6-Octadecanoic acid methylester**	**296.48**	**26.342**	**4.44±0.032**
**21**	**Hexadecanoic acid methyl ester**	**270.45**	**26.808**	**4.89±0.036**
**22**	3-phenyllactic acid	166.17	27.536	1.43±0.040
**23**	Malic acid	134.08	28.013	1.95±0.032
**24**	Pyroglutamic acid	129.04	28.523	1.42±0.040
**25**	Myristic acid	228.37	29.046	0.98±0.016
**26**	Succinic acid	118.09	30.952	2.45±0.040
**27**	Citric acid	192.12	32.085	1.86±0.008
**28**	Phenylacetic acid	136.14	32.583	0.98±0.297
**29**	Phenol,2,4-bis(1,1-dimethylethyl)	278.5	36.604	1.85±0.064
**30**	Phosphoric acid	97.99	39.833	1.45±0.0163
**31**	2-hydroxy isocaproic acid	122.12	42.413	1.95±0.062
**32**	2-hydroxyisovaleric acid	74.08	42.671	1.54±0.013
	**Total percentage identified**			97.27

[#]*The metabolites are listed according to the elution order.*
